# Fungi Burger from Stale Bread? A Case Study on Perceptions of a Novel Protein-Rich Food Product Made from an Edible Fungus

**DOI:** 10.3390/foods9081112

**Published:** 2020-08-13

**Authors:** Coralie Hellwig, Rebecca Gmoser, Magnus Lundin, Mohammad J. Taherzadeh, Kamran Rousta

**Affiliations:** Swedish Centre for Resource Recovery, University of Borås, 50190 Borås, Sweden; rebecca.gmoser@hb.se (R.G.); magnus.lundin@hb.se (M.L.); mohammad.taherzadeh@hb.se (M.J.T.); kamran.rousta@hb.se (K.R.)

**Keywords:** food perceptions, food preferences, novel food, edible filamentous fungi, sustainable food production, resource recovery

## Abstract

The current study aims to assess how a novel fungi product made from the filamentous fungus *Neurospora intermedia*, cultivated on bread residuals, is perceived using questionnaires. Participants were asked to rate characteristic attributes of a fungi burger patty and state their preference when comparing it to Quorn and hamburger patties. The data were analyzed to assess whether gender or age was statistically associated with preference profiles. Neither age nor gender was associated with the preference profiles regarding the comparison of burger patties. Except for age and bitterness, age and gender were also not associated with the preference profiles regarding the sensory characteristics of the fungi burger patty. Most of the participants liked the characteristics of the fungi burger patty. The results indicate that fungi products from waste can become accepted products when information dissemination targets environmental benefits. Moreover, to be commercially accepted, the chewiness and bitterness of the product should be improved. Other improvements should target the overall taste in order to cater to people who prefer meat-based protein sources.

## 1. Introduction

The global population is estimated to increase to ten billion by the year 2050 [1]. This issue goes hand in hand with challenges to provide nutritious food to everyone [2] in sustainable ways that are affordable as well as gentle on the environment and life on earth. The status quo of food production, however, often depletes resources [3] at a time when managing the planet’s resources has never been as important as it currently is [4]. Even though providing the global population with food is increasingly challenging, one-third of all edible food produced for human consumption ends up as waste [5]. This loss of food does not only represent unnecessary CO_2_ emissions and losses of ecological and economic value for both farmers and consumers, but it is additionally linked to ethical concerns as millions of people suffer from undernourishment and/or chronic hunger [6,7].

The limited amount of protein-rich food available in parts of the world [8] contributes to more than one billion people worldwide being unable to meet daily protein requirements [9,10]. This can lead to protein-energy malnutrition, which is a condition that is among the four most common nutritional syndromes [7]. Moreover, the global distribution of food is uneven [6], and this furthers the already raised demand for sustainable, affordable, and accessible high-quality food that meets human protein needs. The status quo of the food industry may not be sustainable in the long run if it relies on animal-based or even plant-based proteins. Providing growing populations with adequate amounts of proteins from animal origin is both expensive and challenging [11]. The production of animal-based proteins generally impacts the environment more severely than the production of plant-based proteins [9].

Nevertheless, producing plant-based sources of protein is sensitive to irregular harvests, land use regulations, and labor intensity [12]. While plant-based proteins may be more sustainable than animal-based ones, a drawback of plant-based proteins is that they might not contain all the essential amino acids or the appropriate ratio of amino acids required in a complete protein source. Moreover, pulses, grains, and seeds may contain antinutritional compounds as part of the natural defense mechanism of the seed, which can interfere with the absorption of nutrients during digestion. For example, the antinutritional compounds protease inhibitors (inhibit proteases from hydrolyzing proteins) and phytic acid (binds and, subsequently, precipitates minerals) in soybeans prevent protein and mineral absorption in the gut and intestines [13].

Biotechnological developments have attempted to address demands to provide appropriately protein-rich food (e.g., [14,15]). Efforts to improve the nutritive value of plants often focus on chemical, physical, and enzymatic treatments, but biological treatments may offer an approach that is gentler to the environment [16]. One way of doing so is to produce fungi-based proteins. Filamentous fungi, comprising a group of eumycetes [17], show promising results in food production when they are cultivated on plants, cereals, and grains. Fungi can improve nutritive values, bioaccessibility, and digestibility of the plants they are cultivated on by synthesizing vitamins, altering the protein and lipid profile, and decreasing antinutritional compounds [18,19,20]. These effects are associated with the metabolic activities of the fungi and their enzymes. Fungi improve nutritive values by producing enzymes that degrade the cell walls of the plant they are cultivated on and so improve the digestibility of available proteins [18]. During solid-state fermentation, (i) proteins are hydrolyzed into amino acids and peptides, of which parts are converted into fungal biomass, (ii) the distribution pattern of fatty acids is altered and the total level of free fatty acids increases, and (iii) antinutritional compounds are broken down [17,18,19]. Furthermore, using *N. intermedia* for solid-state fermentation on stale bread increases the content of dietary fiber, minerals, and vitamins E and D2 [21]. 

Fermentation using edible filamentous fungi does not only show promising indicators to becoming a strategy to improve nutritive values, but it is also a process that can contribute to global nutritional health in a way that is less prone to seasonal changes or climate change, less dependent on fertilizers and pesticides, and requires a lot less space and labor than plant- or animal-based food production [12]. People are currently striving towards behavioral patterns that lower the negative consequences of human activity on the environment, and many have found meaningful ways to answer these calls by changing their dietary habits [22]. For some, this change may manifest in the preference of plant-based products over meat-based ones [23]. Reasons for this can be that the production and consumption of plant-based products are thought to contribute to sustainable food supply, health (both individual and global health), and social justice, and to mediate negative environmental consequences because fewer natural resources are needed in their production [24].

There are numerous plant-based alternatives on the market that cater to those who do not wish to consume meat-based products. However, Gmoser et al. [25] have taken this one step further and demonstrated an innovative way to produce a protein-rich food product, with good nutritional value, made from bread residuals through solid-state fermentation using the edible and generally regarded as safe to eat [26] filamentous fungus *Neurospora intermedia*. Many examples of fermented food products are closely linked to contexts of sociocultural patterns of food preferences, such as fungal fermented soybeans in Indonesia or fermented herring in Sweden. However, fermenting food waste is less common, and fermenting bread residuals using *N. intermedia* to produce a nutrient-enriched food product is novel in Sweden.

In nature, fungi exhibit natural ways to recirculate waste by decomposing organic matter and returning nutrients into the ecosystem [27]. Utilizing this natural process in the production of food creates novel applications for organic waste that can, in turn, contribute to the valorization of waste materials [27]. The benefit of using bread residuals for fermentation is not merely the improvement of its nutrient profile but also the recovery of a plant-based resource that would otherwise go to waste. Minimizing global food waste is one of the targets to achieve sustainability [4]. Bread waste makes up a large proportion of food waste and makes the most significant impact concerning mass, environmental impact, and economic costs in Sweden [28]. The amount of bread waste generated by retailers alone in Sweden is estimated to be roughly 29,870 tons per year [29]. Bread waste can also be an available substrate to use in the production of fungi-based products. Additionally, bread waste is considered safer than food waste from animal origin or food waste that is not yet or no longer safe to eat [30]. In the United States, for example, bread waste is not submitted to obligations such as the boiling before reprocessing [31], which makes it easier to utilize it in the production of new food products. Using bread residuals in the production of food is an innovative opportunity to contribute to efforts taken towards circular economies in which materials are reused (e.g., [32]). Moreover, using bread residuals as a source for fermentation can also eliminate disposal problems associated with waste, lower environmental pollution from waste, and introduce potentially cost-effective processes for the production of valuable compounds due to the low or negative cost of the raw materials [27].

### 1.1. Fungi Cultivation on Bread Residuals

The fermentation process is relatively simple and can be duplicated at bakeries, restaurants, or homes using bread leftovers. Bread waste is widely available, and it is often high in starch and low in protein [33]. Through the fermentation of bread, most of the carbohydrates can be converted into fungal protein, and the nutrients in the bread are concentrated in the final product [21]. In this context, solid bread waste is a potentially valuable and renewable resource, considering the possibilities that lay in the transformation of it into nutritious, high protein food products through fermentation.

Preliminary laboratory research using bread residues resulted in a fungi burger patty, which has promising qualities for human consumption in terms of its texture and nutritive quality [34]. In fact, the protein content of bread can be more than doubled after fermentation for five days using the edible filamentous *N. intermedia* [34]. As earlier mentioned, the production of meat-based food requires large amounts of resources. The technique used by Gmoser et al. [34] eliminates a large part of the supply chain of meat-based products. Fungi-based products fermented on plant-based bread residuals could, thus, be more environmentally and economically sustainable than meat-based products. The idea of reintroducing food waste back into the food chain is innovative and can contribute to social sustainability if the concept finds acceptance.

### 1.2. The Challenge of Perception

There are many products on the market that cater to people who prefer plant-based over meat-based products. However, these products are very rarely explicitly labeled as sourced from resources that would otherwise go to waste. Therein lies the challenge to understand how people perceive fungi-based products made from bread residuals. The level of novelty and its conceptualization will be a critical determinant of whether consumers find a food product made from bread residuals and fungi acceptable to buy and eat. Gaining knowledge of people’s perspectives of fungi-based products from waste is an essential step in establishing such an innovative new source of food.

There are consistent relationships between profiles of traditional food consumers and sociodemographic characteristics such as age, gender, and income. Traditional food consumers who are less open to innovations in the food sector are commonly native and older conservative females, with below-average incomes [35]. In contrast, people who welcome innovations in food products are typically younger and weight-conscious [35]. Moreover, European traditional food consumers tend to be receptive to labels that guarantee the origin of the raw materials, the use of organic raw materials, and new processes that improve food safety [35]. This kind of consumer profiling is particularly useful in product marketing and product development of novel food products. Currently, there is no information on how people perceive fungi-based products made from bread residuals. The objective of this study is, thus, to assess whether age or gender are associated with (i) preferences across different food sources, and (ii) the perceptions of fungi-based products from bread residuals. Furthermore, preferences from a sustainability and economic point of view are also investigated. The benefit of this study is the insight it provides on the fungi burger patty as to which specific product attributes can be customized to specific consumer segments. However, the insight can also be used to communicate and market novel food products that are sourced from sustainable resources.

## 2. Materials and Methods

The aim of the present study is to provide insight into how people perceive the novel fungi-based food product made from bread leftovers. The study was conducted at a tasting event using questionnaires. The main parameters for the investigation of the attributes of the fungi burger patties were age and gender.

### 2.1. Hypothesis

Firstly, it was hypothesized that there is no association between the participants’ age or gender and their preference profiles regarding the fungi, Quorn, and hamburger patties (H_0_^a^). A second hypothesis was that neither the participants’ age nor gender is associated with the preference profiles regarding the rating of the fungi burger patty’s characteristics (H_0_^b^).

### 2.2. Participant Selection

In total, 72 volunteers (*n* = 72), with an equal number of men and women, were recruited to participate in the tasting and answer the questionnaire (Table 1).

The participants were approached at an event in Gothenburg, Sweden, which was marketed as an adventure contest in which teams of two people had six hours to find clues, get around in the city, and complete assignments as fast as they could. The contest was an urban adventure that gave all participants the chance to demonstrate their ability to think, practice logistical and collaboration skills, determination, and flexibility. The assignments were designed to test the participants in various physical, intellectual, fun, and exciting tasks. This venue was perceived to be an opportunity to approach a large group of people of different ages and genders. Given the nature of the event they were approached at, the people who took part in the current study likely had an interest in seeking new and adventurous experiences. There were no factors that would have excluded anyone from participating in this study despite food allergies. All participants completed the tasting and the questionnaire (Appendix A).

### 2.3. Data Collection

The data were collected using an adapted version of descriptive analysis for sensory evaluation because of the richness in detailed information this enabled. A descriptive analysis of new food products allows researchers to identify and describe characteristic attributes of the product and to quantify the perceived intensity of each sensory characteristic [36]. Furthermore, a descriptive analysis not only enables one to study potential consumer preferences but also to assess exactly how one product differs from the established products in sensory terms [37]. In this study, a unique questionnaire was designed to suit the fungi burger patty and the research aim, as suggested by Lundgren [38]. The questionnaire used in the present study was based upon a list of preferences and sensory attributes in order to measure preferences across the three types of patties and relevant characteristics of the fungi burger patty, such as sweetness, bitterness, saltiness, chewiness, texture, and appearance. The number of participants can vary from as little as eight and can be up to one hundred in order to gain enough data to evaluate a food product using a descriptive analysis [36]. In this study, 72 participants were asked to complete a questionnaire that involved stating their age, gender, and diet. Before answering the questionnaire, participants were asked to read an information sheet on the fungi burger patty and how it was made to ensure every participant had the same background knowledge on the product they were going to taste.

In the first section, the participants were asked to taste the fungi burger patty and compare it with two already commercially established products, namely, hamburger patties (beef- and pork-based) and Quorn burger patties (mainly composed of fungal biomass from *Fusarium venenatum*). The main reason for the comparison with these two commercially accepted products was to see how the fungi burger patties made from bread residuals stand against established products through the evaluation of preference profiles. On the questionnaire, the participants were asked to mark the patty option they preferred in terms of most appealing overall taste, most appealing overall texture, which option they preferred for environmental reasons, and which option they would prefer to buy at the grocery store if they were all to cost the same.

In the second section, the participants were asked to rate how they perceived the fungi burger patty’s appearance, bitterness, saltiness, overall taste, chewiness or crispiness, smoothness or sponginess, overall texture, and smell on a scale from one to five. The more satisfactorily the participants experienced a characteristic, the higher they were asked to rate it. Five equaled excellent, four equaled good, three equaled fair, two equaled poor, and one equaled very poor. In order to avoid confusion, the participants were verbally informed that a high rating for the questions on bitterness, saltiness, and sweetness would mean the perfect balance of these characteristics, whereas a low rating would indicate an imbalance.

Participants were blindfolded during the taste comparison of the fungi-, Quorn- and hamburger patties, and were asked to guess which product represented which bite. In between tasting each burger patty option, participants were asked to have a sip of water from their water bottles. The blindfold was then removed, and the participants were asked to fill out the first part of the questionnaire, which was about the preferences across the three burger patties. The participants were offered a taste of the fungi burger patty once more before answering the second part of the questionnaire, which was specifically on the perceptions of the fungi burger patty.

### 2.4. Sample Preparation

Twenty fungi burger patties were produced in accordance with Gmoser et al. [9]. The cultivation of *N. intermedia* was carried out in sterile petri dishes (100 × 20 mm), to which breadcrumbs were added. Then, 15 g total dry weight of bread crumbs were inoculated with spore suspension, and the initial moisture content was adjusted to 40% (on a wet basis (*w*/*w*, wb) with distilled water. Each sample was mixed evenly and then covered with a petri dish lid. Solid-state fermentation was carried out batch-wise for 5 days in a climatic test cabinet (NUVE test cabinet TK 120, Ankara, Turkey) at 90–95% Rh ± 1% at 35 °C under continuous light (3000 lux). The petri dishes were flipped every second day.

Commercial products were also prepared for the tasting event in order to enable a comparison of products: Quorn vegan burger patties with 38% (wet weight) mycoprotein (Quorn^®^, Loughborough, UK) and minced meat with equal amounts of pork and beef made into hamburger patties (120 ± 2 g; ICA, Borås, Sweden). All samples were fried at 110 °C in 5 g of butter for 5 min on each side. Salt was added to the fungi burger patties and hamburger patties, whereas the Quorn burger patties were already seasoned upon purchase. Samples were then frozen and reheated at the tasting event in a microwave for 30 s before being offered to the participants. The samples were cut into 2 cm pieces and served on three plates: one with fungi burger patty pieces, one with hamburger pieces, and one with Quorn burger pieces. Additional information on the samples is presented in Appendix B. When served, the temperature of the pieces had cooled down to room temperature. One piece of each burger patty option was served to every participant in random order on sticks.

### 2.5. Data Analysis

The raw data collected through the questionnaire were statistically analyzed using cross-tabulation and chi-square analysis for association in order to assess whether or not gender or age were associated with preference profiles at a significance level of 5%. While a chi-square test cannot prove that two samples of groups are the same, it will prove whether or not the hypothesis can be rejected [39]. The analysis was done using MINITAB^®^ [40]. The association of age or gender across the first and second part of the questionnaire was also investigated (i.e., the participants’ preferences across the three burger patty options in the first part and the tasting experience of the fungi burger patty in the second part). The overall preference profiles of each question were also analyzed to assess the findings of the taste experience of the fungi burger and the participants’ preferences as a group.

## 3. Results and Discussion

The results of the comparison across the fungi, Quorn, and hamburger burger patties proved that neither gender nor age was statistically associated with preference profiles in terms of overall taste, overall texture, environmental reasons, or cost. While the fungi burger patty’s taste and texture might need to be improved in order to get more people to prefer it over the Quorn and hamburger options, the participants preferred it most for environmental reasons and would also purchase it if the cost was the same as the other options. Gender was not statistically associated with the preference profiles in any of the fungi burger patty’s characteristics. However, age proved to be statistically associated with the preference profiles on the bitterness of the fungi burger patty (*p* = 0.041) at a significance level of 5%, but not at 2.5% (*p* > 0.025). The majority of the participants liked each characteristic of the fungi burger patty.

### 3.1. Comparison with Established Products

Even though gender was found to be almost associated with the preference profiles regarding the overall texture (*p* = 0.054), the hypothesis that neither age nor gender would be associated with the preference profiles regarding the fungi, Quorn, and hamburger patties (H_0_^a^) proved to be true. In total, most participants were able to correctly identify which burger patty option they tasted when blindfolded. However, roughly 15% of the participants mistook the Quorn burger patty for the fungi burger patty. Given that both patties are fungi-based, this finding indicates that the use of bread residuals in the cultivation process provides a distinct flavor to the fungi burger patty.

**Figure 1:**
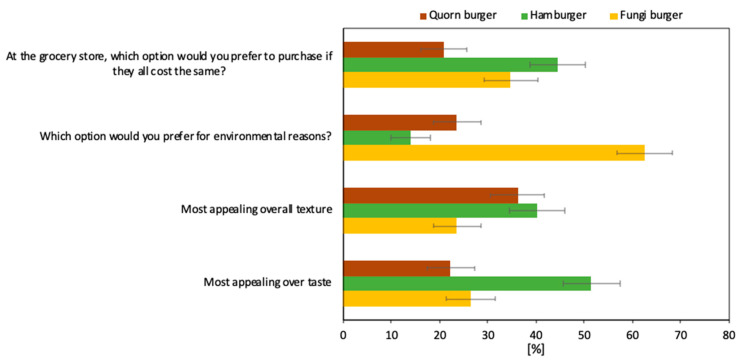
Distribution of the participants’ (*n* = 72) preferences across burger patty options. Error bars represent one standard uncertainty.

Most participants indicated that they would purchase the hamburger option for most appealing taste and if all three burger patty options cost the same. The fungi burger scored second highest in these contexts, followed by the Quorn option. Most participants indicated that they prefer the fungi burger patty for environmental reasons, whereas the Quorn and hamburger options were less often preferred. Lastly, most participants preferred the texture of the hamburger over the other two options. The Quorn burger received the second-highest score in this respect, and the fungi burger patty’s texture was least often preferred. The distribution of preferences across the three burger patty options are displayed in Figure 1.

When asked to state their preference across the three options in terms of *most appealing overall taste*, 26% of the participants preferred the fungi burger, while 51% preferred the hamburger patties and 22% preferred the Quorn patties. Interestingly, providing information on how a food product is manufactured may influence how the taste of that product is evaluated [41]. Knowledge of the production process of the fungi burger patty, disseminated both verbally and through an information sheet, may have had a psychological effect on the perception of the product.

In terms of *most appealing overall texture*, 24% of the participants preferred the fungi burger patties, while 40% preferred the hamburger and 36% the Quorn burger patties. There was no significant association of age with the preference profiles regarding the most appealing overall texture across the three burger patties. However, the distribution of preferences across the burger patty options was relatively even for women, while men preferred the hamburger over the other options.

When asked which option the participants preferred for *environmental reasons*, 63% of the participants favored the fungi burger patty, while 24% favored the Quorn burger patty. Moreover, 14% of the participants preferred the hamburger patty. This indicates that there might be a disconnect in the spread of research findings to the broader public, and that information on the environmental impact of meat production might not reach everyone across society. However, even when it does, the knowledge may have an additive effect rather than drive people’s food product preferences. Most participants preferred the fungi burger patty for environmental reasons. This may have resulted from the successful information provision before the participants responded to the questionnaire. The information provided pointed out that bread residuals were used in the production of the fungi burger patty. Quorn also markets their fungi-based product as an environmentally sustainable meat alternative, and the participants were verbally informed of this. However, written information on how Quorn burger patties are produced was not provided to the participants as this information is hard to obtain. Given that the current study did not assess the impact of information provision on preference profiles, future research might gain interesting insights when studying whether information on production processes and environmental, economic, and social consequences for each of the three burger patty options impacts preference profiles.

Environmental attitudes can moderately influence the acceptance of innovative food technologies [42]. Even though people are often aware that meat production negatively impacts the environment [43], as much as 14% of the participants preferred hamburger patties for environmental reasons. The notion of naturalness might also influence the way people perceive innovative food technologies [44] because some people may consider tampering with nature a predictor for risk [45]. Research has found that people perceive products and food technologies with tangible benefits as less harmful than those without obvious benefits [44]. In this context, it might be interesting to study how people who value organic food products perceive fungi foods in general, as well as fungi foods based on bread residuals.

If all the three burger patties were to cost the same at the grocery store, 35% of the participants said that they would purchase fungi burger patties, while 21% would purchase Quorn burger patties and 44% hamburger patties. This result indicates that the fungi burger would compete well with other commercially established meat alternatives if its price is similar. However, the price of the fungi burger patty may need to be lower than that of their meat-based counterparts in order to make it attractive to people. This is in line with Zhu and van Ierland [46], who found that the substitution effect of novel protein food products depends on their price relationship to alternative products and that lowering their price aids their replacement of meat-based protein sources. Furthermore, the environmental and potential health benefits of novel protein products from waste need to be well communicated by stimulating the environmental concerns of people and providing them with information about the environmental performance of fungi-based food products in terms of their contribution to sustainable consumption patterns [46].

The perceived benefits of innovative food technologies can importantly influence whether or not a new food product will be accepted [44]. In the context of the fungi burger, the results indicate that the participants associated environmental benefits with the fungi product and that they might purchase it if doing so would not impact them financially. This may be different for other segments of people that the fungi burger patty may cater to, outside the participant group. However, perceived risk, such as the risk posed by additional financial strain, is negatively correlated with people’s willingness to purchase a product [47].

### 3.2. Statistical Associations across the Comparison Section

Several statistical associations were found regarding the results of the participants’ preferences across the three burger patty options (Appendix C). For example, the preference profile regarding which one of the three burger patty options the participants preferred for *environmental reasons* was found to be statistically associated with which burger option they preferred if all three options *cost* the same (*p* = 0.001). This result indicates that participants who preferred the fungi burger patty for environmental reasons would also prefer to buy it at the grocery store if all three options cost the same. Despite hamburgers being less environmentally friendly than the other two options, participants who preferred hamburger patties for environmental reasons would also purchase it at the grocery store. This indicates that such knowledge may not be sufficiently disseminated. However, this result may also indicate that factors other than environmental awareness, such as cost, influence people’s preferences. Nevertheless, the participants who preferred the Quorn burger patty for environmental reasons would buy either the hamburger or Quorn burger patty (equal distribution) at the grocery store if they cost the same.

Furthermore, the preferences in terms of the *most appealing overall taste* were statistically associated with the preference profiles regarding which option the participants would purchase if they were to *cost* the same (*p* = 0.000). Promising results for the fungi burger patty also showed that those who found the fungi burger patty’s taste most appealing also indicated that they would prefer to buy it even when the hamburger and Quorn burger patties would cost the same at the grocery store. Those who found the fungi burger most appealing indicated that they would purchase the hamburger less often than expected. Furthermore, those who preferred the overall taste of the hamburger indicated that they would be less likely to purchase the fungi burger patty.

The burger patty option the participants would choose for *environmental reasons* was also statistically associated with the preference profiles regarding which option they would choose to purchase if all three options were to *cost* the same (*p* = 0.001). As expected, those who preferred the fungi burger patty for environmental reasons also indicated that they would purchase this option if it were to cost the same as the others. However, the environment does not seem to be the only factor informing the participants’ choices, given that many of the participants who preferred the fungi burger patties for environmental reasons indicated that they would actually choose the hamburger option if the cost were the same. A similar pattern was observed for participants who preferred the Quorn burger patty for environmental reasons. Nevertheless, the consumption of novel protein foods is increasingly trendy in Europe due to the heightened awareness of health, food safety, and environmental concerns associated with meat-based protein [46]. Moreover, those who preferred the hamburger patty for environmental reasons would purchase the more environmentally sound fungi burger less often and, instead, purchase the hamburger option if they were to cost the same. In the context of the costs of alternative protein-rich food products, however, increasing incomes in Europe may spur trends and influence the willingness of people to pay for sustainable and environmentally beneficial products [46].

Furthermore, the participants’ preferences regarding the *most appealing overall taste* were found to be statistically associated with the burger patty option they would prefer for *environmental reasons* (*p* = 0.015). The majority of the participants who preferred the taste of the fungi burger patty would also choose this option for environmental reasons. The participants who preferred the taste of the Quorn burger patties equally preferred, more or less, the Quorn or fungi burger option for environmental reasons.

### 3.3. Perceptions on the Fungi Burger Patty

**Figure 2:**
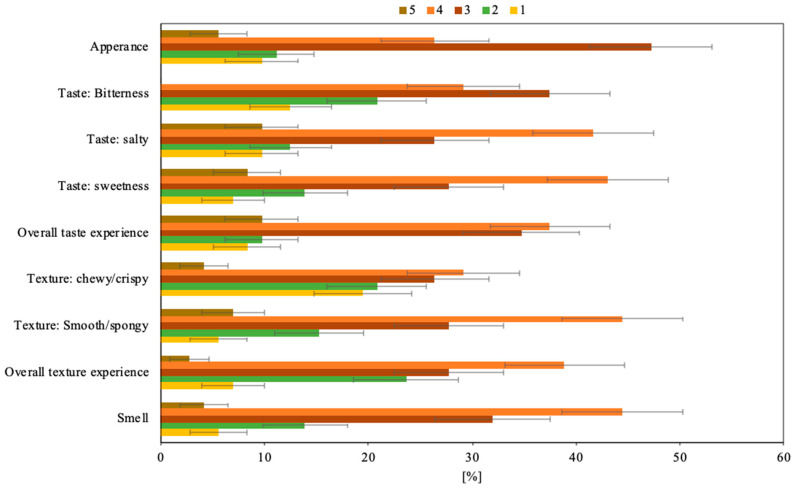
Distribution of the participants’ (*n* = 72) preferences on the characteristics of the fungi burger. The five-point hedonic rating scale of the characteristics is represented in the bars that ascend from excellent (5) to very poor (1). Error bars represent one standard uncertainty.

Neither age nor gender was statistically associated with the preference profiles in this section of the questionnaire. The only exception was that age was associated with preferences regarding the bitterness of the fungi burger patty. The second hypothesis that neither age nor gender is associated with the preference profiles regarding the participants’ ratings of the fungi burger patty’s characteristics (H_0_^b^) proved, thus, false. When participants evaluated the fungi burger in more detail, the overall preference was rated as good except in terms of appearance and bitterness, where the majority of participants rated the fungi burger patty as fair. Few participants rated each of the characteristic attributes as either very poor or excellent (Figure 2).

The appearance of a food product, alongside its flavor, nutritional properties, and texture, are principal characteristics defining food quality [48]. In terms of *appearance*, the majority of the participants rated this characteristic as fair or good. One reason for this result can be that the fungi burger patties were cut into bite-size pieces for the tasting, which made it harder for the participants to evaluate its appearance. The results indicate that the majority of the participants liked the fungi burger patty’s appearance. However, the results also indicate that the perception of this characteristic could also be improved. Future research may gain valuable information from qualitatively assessing the way people think its appearance should develop. This information may be constructive in efforts targeting the fungi burger patty’s acceptance and to encourage people to consume it.

The taste of filamentous fungi fermented food can be influenced by several amino acids [49]. As an example, glutamic acid tastes umami or savory [50], which is characteristic of, for example, cooked meat. The participants were asked to rate the overall taste, as well as the characteristics of bitterness, saltiness, and sweetness, to better understand how the taste of the fungi burger patties are perceived. Regarding the *overall taste experience*, most of the participants rated this characteristic as good or fair. The distribution of the participants’ preferences regarding the fungi burger patty’s overall taste is in line with research that has described the taste of products made of edible filamentous fungi, such as *N. intermedia*, as generally pleasant [51].

Many participants rated the *bitterness* of the fungi burger patty as fair, while others rated it as either well-balanced or slightly imbalanced, and a few as very imbalanced. Given that a high rating of this characteristic would indicate a perfect balance of bitterness, the result indicates that most of the participants did not seem to perceive the fungi burger patty as overly bitter. While gender was not statistically associated with the preference profiles regarding the bitterness of the fungi burger patty (*p* = 0.093), age was (*p* = 0.041). Participants between 30–40 and 41–60 years of age perceived the bitterness as fair. However, younger participants, who were between 19–29 years of age, liked this characteristic better.

The *saltiness* of the fungi burger patty was rated by most participants as well-balanced or fair. The same was true for the *sweetness* of the fungi burger, which most participants also rated as well-balanced or fair. In order to better understand how people perceive the bitterness, sweetness, and saltiness of food products like the fungi burger patty, it might be insightful to utilize qualitative elements in future research. This is because follow-up questions could then investigate why some people rate particular characteristics as off-balance, and in what way they think the product could be improved.

Texture is among the principal characteristics defining food quality [48]. Moreover, the texture of a food product is also generally recognized as an important factor influencing whether or not a food product will be accepted [52]. This is especially important for meat alternatives, like the fungi burger patty, because texture is the most challenging obstacle in growing wide acceptance [53]. The majority of the participants rated the *overall texture experience* of the fungi burger patty as good, and many rated it as either fair or poor. The texture of filamentous fungi, such as *N. intermedia*, is thought to be an advantage in food production [34]. This is because such fungi produce filaments that are much like those in meat, with a texture similar to that of lean meat [54]. The fungi burger patty has a similar texture to that of soybean burger patties, but the final texture can be improved to meet the expectations of the people it caters to through the addition of minor ingredients [34].

In order to gain additional insight into how the participants perceived the textural characteristics of the fungi burger patties, they were asked to rate the *smoothness or sponginess* of it. The majority of participants rated this characteristic as good, while many found it to be fair, and a few to be either excellent, poor, or very poor. Insight was also gathered on the *chewiness or crispiness* of the fungi burger patties. The participants rated this textural characteristic relatively evenly as good, fair, poor, and very poor, while a few rated it excellent. The results indicate that the texture characteristics of the fungi burger patty were generally positively perceived. However, the results also indicate that chewiness or crispiness should be improved in order to gain widespread positive perceptions of this characteristic. In order to address this, future research on people’s perceptions of the fungi burger patty could quantitatively investigate the way people think the product should be developed.

Lastly, the majority of participants rated the *smell* of the fungi burger patty as good or fair. This is in line with research that has reported the smell of products made of edible filamentous fungi, such as *N. intermedia*, to be generally pleasant [51]. The fungi burger patties that are cultivated on bread residuals predominately smell of mushrooms but also have a nutty and fruity smell [34], which seems to be perceived as pleasant by many participants.

### 3.4. Statistical Associations across the First and Second Parts of the Questionnaire

The preference profile of the *most appealing overall taste* across the three burger patty options was statistically associated with the ratings of the *overall taste experience* of the fungi burger patty (*p* = 0.003) (Appendix C). Participants who preferred the fungi burger or hamburger had different appreciation profiles for their perceived overall taste of the fungi burger patty. This indicates that participants who preferred the fungi burger patty over the other burger patties rated the fungi burger patty’s overall taste significantly more often as good than the hamburger and Quorn burger patties, which were mostly rated as fair. Furthermore, the participants that preferred the fungi burger patty to the other options shifted their taste experience of the fungi burger from fairly good and good to very good and excellent.

Lastly, the preferences of which patty option the participants would purchase if they were all to *cost* the same were also statistically associated with the preference profiles regarding the *overall taste experience* (*p* = 0.014). Participants who preferred the hamburger patty if all three burger patty options were to cost the same rated the overall taste experience of the fungi burger patty lower than the participants who preferred the Quorn and fungi burgers in this respect. The taste experience for participants who prefer meat seems to differ from those open to other options. It would thus be interesting to investigate whether the participants who preferred to purchase hamburger patties would rate the taste of the fungi burger patty differently if they did not taste the hamburger beforehand.

### 3.5. Limitations

One of the limitations of using chi-square analysis in this study is the sample size of *n* = 72 and the impact that this had on multiple comparisons of the data. An association may be statistically associated even though the findings are not substantively significant. In this study, there was a 5% probability of making at least one type 1 error, i.e., rejecting at least one true hypothesis. As an example, gender was found to be almost statistically associated with the preference profiles regarding the overall texture (*p* = 0.054) across the three burger patty options. This association may be interpreted as a reasonably strong association. However, with a significance level of 5% in multiple comparisons, one out of 20 associations will result in a significant effect just by chance. Similarly, this could have been true for the preference profiles on the bitterness of the fungi burger patty to which age was statistically associated (*p* = 0.041).

Moreover, a descriptive analysis of the sensory characteristics of food products is typically conducted using a panel of well-trained individuals who have developed an analytical frame of mind to sense specific characteristics, such as taste, texture, and smell [36,55]. However, an adapted version of a descriptive analysis was performed in order to better understand how people from across society experience the fungi burger and to assess their preferences regarding the different burger patties. This strategy was employed because the current study aimed to understand how potential consumers rate the product as opposed to how trained professionals would.

## 4. Conclusions

The hypothesis that neither age nor gender would be associated with the preference profiles regarding the fungi, Quorn, and hamburger patties (H_0_^a^) proved to be true. The second hypothesis that neither age nor gender is associated with the preference profiles regarding the participants’ ratings of the fungi burger patty’s characteristics (H_0_^b^) proved to be false, given that age proved to be statistically associated with the preference profiles on the bitterness of the fungi burger patty (*p* = 0.041) at a significance level of 5%. The majority of the participants liked the characteristics of the fungi burger, and only a few perceived certain characteristics very positively or very negatively. The results suggest that the fungi burger patty’s chewiness and bitterness can be improved and that this can be done most constructively if future research utilizes a qualitative or mixed-methods design to assess in what way participants think the product should be developed. In addition, other improvements should target the fungi burger patty’s overall taste for it to cater to people who prefer meat-based protein sources. Participants who preferred the fungi burger patty over the other patties actually indicated that they would purchase the fungi burger at the grocery store even if it cost the same as hamburgers. Some of the participants who preferred the taste of the hamburger or Quorn burger also indicated that they preferred the fungi burger patty for environmental reasons. The findings indicate that creating new products from waste might find acceptance when information about such products and environmental benefits are clearly communicated.

## Figures and Tables

**Table 1:** Profile of the participants.

Gender	Age Group 1 (19–29)	Age Group 2 (30–40)	Age Group 3 (41–60)	Total
Female	56%	44%	52%	50%
Male	44%	56%	48%	50%
Combined	25%	35%	40%	100%

**Figure A1:**
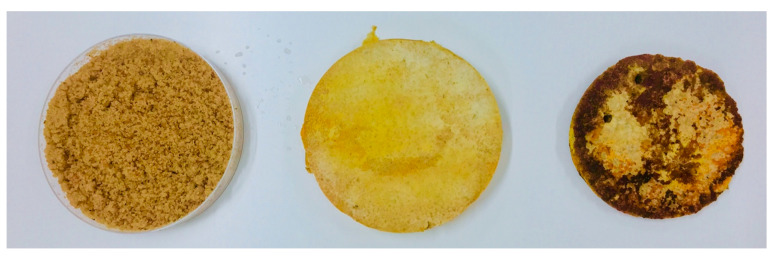
The fungi burger patty before fermentation, after fermentation, and after frying the fermented product (from left to right).

**Figure A2:**
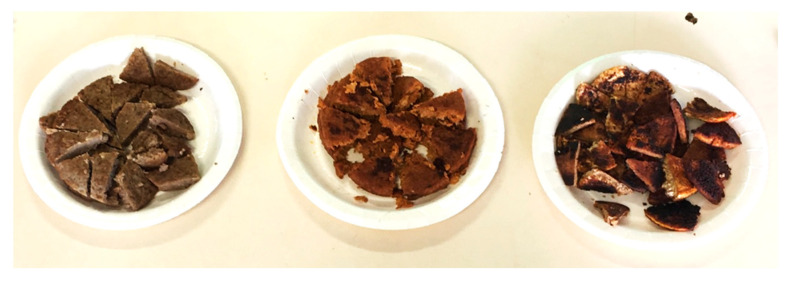
Samples of the hamburger, Quorn, and fungi (from left to right), the way they were served to participants.

**Figure A3:**
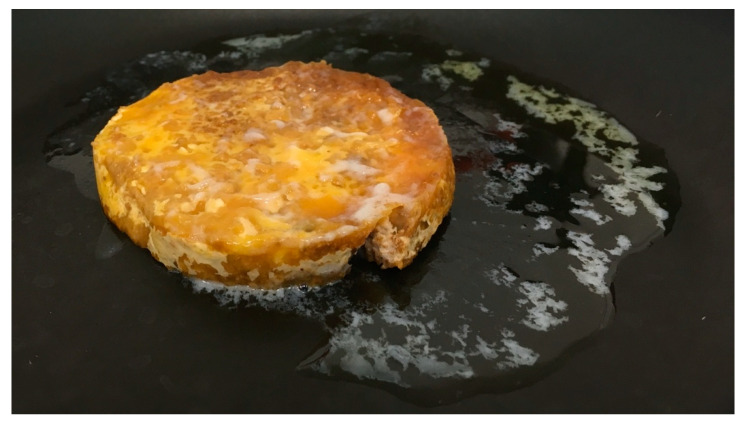
Fried fungi burger patty.

## Appendix A. Questionnaire

We are developing a fungal burger made by fermented stale sourdough bread by edible fungi. We are interested in YOUR first impression and experience on this product. Your contribution is very valuable and unique to us and you will contribute with important data to our research. Your participation is, of course, voluntary and responses are anonymous. Thank you for taking a moment to answer the following questions:

1. **Please specify your gender**
  o Woman
  o Man
  o Non-bindery
2. **Please specify your age**
  o 0–18
  o 19–29
  o 30–40
  o 40–60
  o >60
3. **Please specify your diet**
  o Vegan
  o Vegetarian
  o Omnivore (eating everything)
  o Other diet
4. **Please let us know your opinion and mark which one of the products you just tasted most suits the following aspects:**

**Table:**

	*Fungi Burger*	*Hamburger*	*Quorn Burger*
* Most appealing overall taste*	O	O	O
*Most appealing overall texture*	O	O	O
*Which option would you prefer for environmental reasons?*	O	O	O
*At the grocery store, which option would you prefer to purchase if they all cost the same?*	O	O	O

The next questions target the fungal burger, so feel free to help yourself to another piece before answering the next section.

5. **Based on your experience, please mark the alternative that describes the following sensory attributes of the fungi burger:**

**Table:**

	*Excellent*	*Good*	*Fair*	*Poor*	*Very Poor*
*Smell*	O	O	O	O	O
*Overall Texture experience*	O	O	O	O	O
*Texture: Smooth/spongy*	O	O	O	O	O
*Texture: chewy/crispy*	O	O	O	O	O
*Overall taste experience*	O	O	O	O	O
*Taste: sweetness*	O	O	O	O	O
*Taste: salty*	O	O	O	O	O
*Taste: bitterness*	O	O	O	O	O
*Appearance*	O	O	O	O	O

## Appendix B

Pictures of the fungi burger patty, and the burger patties offered during the tasting

## Appendix C

**Table A1:** Chi-square test for association. Rows—age group; columns—taste (bitterness of the fungi burger patty) *.

	1	2	3	4	All	Cell Contents
**Age (19–29)**	1	6	4	7	18	Count
	−1.25	2.25	−2.75	1.75		Residual
	−1.029	1.508	−1.546	1.048		Adjusted residual
	0.6944	1.35	1.1204	0.5833		Contribution to Chi-square
**Age (30–40)**	1	7	12	5	25	Count
	−2.125	1.792	2.625	−2.292		Residual
	−1.591	1.092	1.342	−1.248		Adjusted residual
	1.445	0.6163	0.735	0.7202		Contribution to Chi-square
**Age (41–60)**	7	2	11	9	29	Count
	3.375	−4.042	0.125	0.542		Residual
	2.452	−2.391	0.062	0.286		Adjusted residual
	3.1422	2.7037	0.0014	0.0347		Contribution to Chi-square
**All**	9	15	27	21	72	

* This characteristic was assessed using a five-point hedonic scale. However, no participant rated this characteristic as excellent (5).

**Table A2:** Chi-square test for association. Rows—which option would you prefer for environmental reasons; columns—at the grocery store, which option would you prefer to purchase if they all cost the same.

	Fungi Burger	Hamburger	Quorn Burger	All	Cell Contents
**Fungi burger**	23	14	8	45	Count
	15.625	20	9.375		Residual
	1.866	−1.342	−0.449		Adjusted residual
	3.481	1.8	0.202		Contribution to Chi-square
**Hamburger**	0	9	1	10	Count
	−3.472	4.556	−1.083		Residual
	−2.485	3.124	−0.909		Adjusted residual
	3.472	4.669	0.563		Contribution to Chi-square
**Quorn burger**	2	9	6	17	Count
	−3.903	1.444	2.458		Residual
	−2.275	0.807	1.68		Adjusted residual
	2.58	0.276	1.706		Contribution to Chi-square
**All**	25	32	15	72	

**Table A3:** Chi-square test for association. Rows—most appealing overall taste; columns—overall taste experience.

	1	2	3	4	5	All	Cell Contents
**Fungi burger**	0	0	3	11	5	19	Count
	−1.583	−1.847	−3.597	3.875	3.153		Residual
	−1.532	−1.667	−2.02	2.14	2.846		Adjusted residual
	1.583	1.847	1.961	2.107	5.381		Contribution to Chi-square
**Hamburger**	6	6	15	9	1	37	Count
	2.917	2.403	2.153	−4.875	−2.597		Residual
	2.488	1.912	1.066	−2.374	−2.067		Adjusted residual
	2.759	1.605	0.361	1.713	1.875		Contribution to Chi-square
**Quorn burger**	0	1	7	7	1	16	Count
	−1.333	−0.556	1.444	1	−0.556		Residual
	−1.368	−0.532	0.86	0.586	−0.532		Adjusted residual
	1.333	0.198	0.376	0.167	0.198		Contribution to Chi-square
**All**	6	7	25	27	7	72	

**Table A4:** Chi-square test for association. Rows—most appealing overall taste; columns—which option would you prefer for environmental reasons.

	Fungi Burger	Hamburger	Quorn Burger	All	Cell Contents
**Fungi burger**	16	1	2	19	Count
	4.125	−1.639	−2.486		Residual
	2.278	−1.267	−1.565		Adjusted residual
	1.433	1.018	1.378		Contribution to Chi-square
**Hamburger**	20	9	8	37	Count
	−3.125	3.861	−0.736		Residual
	−1.522	2.633	−0.409		Adjusted residual
	0.422	2.901	0.062		Contribution to Chi-square
**Quorn burger**	9	0	7	16	Count
	−1.000	−2.222	3.222		Residual
	−0.586	−1.822	2.151		Adjusted residual
	0.100	2.222	2.748		Contribution to Chi-square
**All**	45	10	17	72	
